# *hTERT, MYC* and *TP53* deregulation in gastric preneoplastic lesions

**DOI:** 10.1186/1471-230X-12-85

**Published:** 2012-07-06

**Authors:** Tanielly Cristina Raiol Silva, Mariana Ferreira Leal, Danielle Queiroz Calcagno, Carolina Rosal Teixeira de Souza, André Salim Khayat, Ney Pereira Carneiro dos Santos, Raquel Carvalho Montenegro, Silvia Helena Barem Rabenhorst, Mayara Quaresma Nascimento, Paulo Pimentel Assumpção, Marília de Arruda Cardoso Smith, Rommel Rodríguez Burbano

**Affiliations:** 1 Laboratório de Citogenética Humana, Instituto de Ciências Biológicas, Universidade Federal do Pará, CEP: 66073-000, Belém, PA, Brazil; 2 Disciplina de Genética, Departamento de Morfologia e Genética, Universidade Federal de São Paulo, Rua Botucatu 740, CEP 04023-900, São Paulo, SP, Brazil; 3 Unidade de Alta Complexidade em Oncologia, Hospital Universitário João de Barros Barreto, Universidade Federal do Pará, CEP: 60673-000, Belém, PA, Brazil; 4 Laboratório de Genética Molecular, Departamento de Patologia e Medicina Forense, Escola de Medicina, Universidade Federal do Ceará, CEP: 60020-181, Fortaleza, CE, Brazil

**Keywords:** hTERT, MYC, TP53, Gastric carcinogenesis, Precancerous lesions

## Abstract

**Background:**

Gastric cancer is a serious public health problem in Northern Brazil and in the world due to its high incidence and mortality. Despite the severity of the disease, more research is needed to better understand the molecular events involved in this intestinal-type gastric carcinogenesis process. Since precancerous lesions precede intestinal-type gastric cancer, here, we evaluated the *hTERT*, *MYC,* and *TP53* mRNA and protein expression, as well as *TP33* copy number, in gastric preneoplastic lesions.

**Methods:**

We evaluated 19 superficial gastritis, 18 atrophic gastritis, and 18 intestinal metaplasia from cancer-free individuals of Northern Brazil. Quantitative reverse transcription PCR was used to analyze the mRNA expression and immunohistochemical methods were used to assess protein immunoreactivity in tissue samples. The number of *TP53* gene copies was investigated in gastric diseases by quantitative PCR.

**Results:**

We observed hTERT, MYC, and p53 immunoreactivity only in intestinal metaplasia samples. The immunoreactivity of these proteins was strongly associated with each other. A significantly higher MYC mRNA expression was observed in intestinal metaplasia compared to gastritis samples. Loss of *TP53* was also only detected in intestinal metaplasia specimens.

**Conclusions:**

We demonstrated that hTERT, MYC, and TP53 are deregulated in intestinal metaplasia of individuals from Northern Brazil and these alterations may facilitate tumor initiation.

## Background

Gastric cancer is the fourth most common cancer and the second leading cause of cancer-related death worldwide [1]. In Northern Brazil, gastric cancer is the second most frequent neoplasia among males and the third in females [2]. Some histopathological lesions precede well-differentiated or intestinal-type gastric cancer. These neoplasia types progress through a number of sequential steps beginning with superficial gastritis, followed by chronic atrophic gastritis, intestinal metaplasia, intraepithelial neoplasia, and finally, carcinoma [3]. Although this neoplasia is a serious public health problem in Northern Brazil and in the world, little is known about the molecular events involved in the gastric carcinogenesis process. A better understanding of the critical alterations implicated in tumor initiation is necessary to reduce the mortality rates through early diagnosis and treatment.

Cell immortalization has been reported as an important event in carcinogenesis. This process requires activation of telomerase, an enzyme essential for stabilizing telomere length. Telomerase activation is described in about 90% of human cancers, while most normal tissues contain inactivated telomerase [4]. In the absence of genome checkpoint functions, telomere dysfunction accelerates genomic instability, facilitating tumor initiation [5]. This genomic instability caused by telomere dysfunction occurs in the early stages of carcinogenesis, before telomerase activation. Subsequently, telomeres in these initiated cells undergo further progressive shortening, generating rampant chromosomal instability and threatening cell survival. Telomerase activation occurs at this stage to stabilize the genome and confer unlimited proliferative capacity upon the emerging and evolving cancer cell. Therefore, cells that have acquired telomerase activity can obtain the capacity for cancer progression [6].

Transcriptional regulation of *hTERT* (the catalytic subunit of telomerase) gene is the major mechanism for cancer-specific activation of telomerase. Several factors have been reported to directly or indirectly regulate the *hTERT* promoter, including cellular transcriptional activators, such as MYC, as well as the repressors, such as p53 (see review [6]).

The *MYC* proto-oncogene has been described as a key in the gastric carcinogenic process [7]. MYC protein has an effect on about 15% of genes in the human genome [8]. MYC activates several genes involved in cell cycle regulation, metabolism, ribosome biogenesis, protein synthesis, and mitochondrial function, while it consistently represses genes involved in cell growth arrest and cell adhesion, and also has a direct role in the control of DNA replication [9]. Among the *MYC* target genes are *hTERT* as well as *TP53*[9].

*TP53* is a key tumor suppressor gene in the carcinogenesis process [10] acting in the DNA damage response and apoptosis, as well as a regulator of cell metabolism [11]. *TP53* somatic alteration is described in approximately 50% of human cancers, including gastric cancer [10]. Moreover, the loss of the *TP53* locus is a common finding in gastric neoplasias of individuals from Northern Brazil [12].

The aim of the present study was to determine whether *hTERT*, *MYC,* and *TP53* mRNA expression, as well as their protein products, are deregulated in gastric preneoplastic lesions from cancer-free individuals of Northern Brazil. The number of *TP53* gene copies was also evaluated in gastric diseases.

## Results

**Figure 1:**
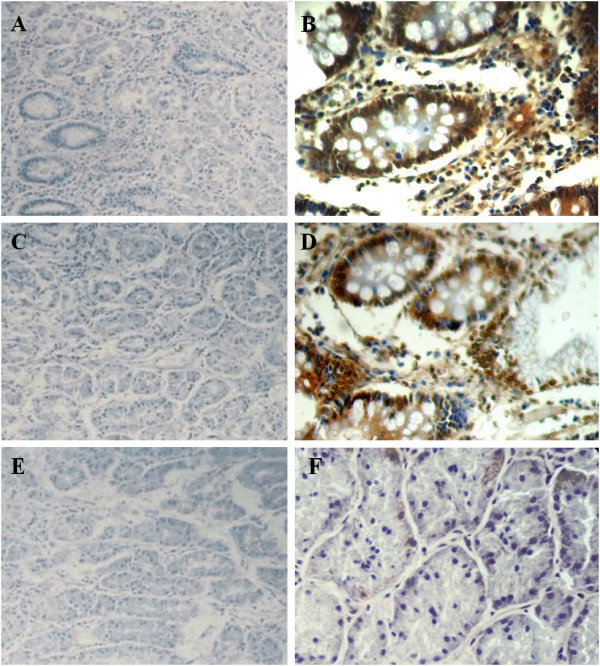
**Immunostaining of hTERT, MYC and p53.** (**A**) Absence of hTERT staining in atrophic gastritis (100×); (**B**) hTERT immunoreactivity in intestinal metaplasia (400×); (**C**) Atrophic gastritis without MYC immunoreactivity (100×); (**D**) MYC immunopositivity in intestinal metaplasia (400×); (**E**) Absence of p53 immunoreactivity in atrophic gastritis (100×); (**F**) negative control (400×).

hTERT, MYC, and p53 immunostaining was only detected in intestinal metaplasia samples (Figure 1, Table 1). The frequency of hTERT (χ^2^ = 19.243, df = 2, p < 0.001, by Pearson Chi-square, V = 0.592), MYC (χ^2^ = 19.243, df = 2, p < 0.001, V = 0.592), and p53 (χ^2^ = 13.844, df = 2, p = 0.001, V = 0.502) immunoreactivity differed among groups (Table 1). Using the Bonferroni correction, a series of Fisher exact tests demonstrated that the frequency of hTERT (p = 0.001, OR = 1.9), MYC (p = 0.001, OR = 1.9), and p53 (p = 0.01, OR = 1.58) immunoreactivity was higher in intestinal metaplasia than in superficial gastritis. Intestinal metaplasia samples also presented a higher frequency of hTERT (p = 0.001, OR = 1.8), MYC (p = 0.001, OR = 1.8), and p53 (p = 0.008, OR = 1.5) immunoreactivity compared to atrophic gastritis specimens. hTERT was strongly associated with MYC (χ^2^ = 40.086, df = 1, p < 0.001, V = 0.854, OR = 0.024) and p53 (χ^2^ = 39.566, df = 1, p < 0.001, V = 0.848, OR = 0.041) immunoreactivity. MYC and p53 immunoreactivity was also strongly associated (χ^2^ = 25.638, df = 1, p < 0.001, V = 0.683, OR = 0.073). Five of 18 (27.8%) intestinal metaplasia samples presented immunoreactivity for the three studied proteins.

**Table 1:** **Clinicopathological characteristics, *MYC*, *hTERT *and *TP53 *expression*, *and *TP53 *copies in gastric samples**

	**Superficial gastritis**	**Atrophic gastritis**	**Intestinal metaplasia**
**Gender**			
Male [N (%)]	12 (63.2)	11 (61.1)	12 (66.7)
Female [N (%)]	7(36.8)	7 (38.9)	6 (33.3)
**Alcohol consumption**			
Negative [N (%)]	10 (52.6)	10 (55.6)	12 (66.7)
Positive [N (%)]	9 (47.4)	8 (44.4)	6 (33.3)
**Cigarette smoking**			
Negative [N (%)]	10 (52.6)	12 (66.7)	9 (50)
Positive* [N (%)]	9 (47.4)	6 (33.3)	9 (50)
**H. pylori infection**			
Negative [N (%)]	7 (36.8)	5 (27.8)	1 (5.6)
Positive [N (%)]	12 (63.2)	13 (72.2)	17 (94.4)
***hTERT*mRNA expression**			
RQ (Mean ± SD)	1.092 ± 0.61	0.821 ± 0.47	1.184 ± 0.55
***MYC*mRNA expression**			
RQ (Mean ± SD)	0.852 ± 0.51**	0.873 ± 0.28**	1.901 ± 0.49
***TP53*mRNA expression**			
RQ (Mean ± SD)	0.997 ± 0.59	0.847 ± 0.79	1.021 ± 0.61
**hTERT immunoreactivity**			
Negative [N (%)]	19 (100)	18 (100)	10 (55.6)
Positive [N (%)]	0 (0)**	0 (0)**	8 (44.4)
**MYC immunoreactivity**			
Negative [N (%)]	19 (100)	18 (100)	10 (55.6)
Positive [N (%)]	0 (0)**	0 (0)**	8 (44.4)
**p53immunoreactivity**			
Negative [N (%)]	19 (100)	18 (100)	12 (66.7)
Positive [N (%)]	0 (0)**	0 (0)**	6 (33.3)
***TP53*copies**			
2 copies [N (%)]	18 (100)	18 (100)	15 (83.3)
1 copy [N (%)]	0 (0)	0 (0)	3 (16.7)***

RQ: relative quantification.

*Patients smoking one or more cigarettes per day (range 1–20).

**Significantly different from intestinal metaplasia group.

***Gastritis was significantly different from intestinal metaplasia group.

We observed a significant difference in *MYC* (F = _2,52_ = 41.172, p < 0.001, by ANOVA test, η^2^ = 0.613) mRNA expression among the studied groups (Table 1). Tukey post-hoc analyses revealed that *MYC* mRNA expression was higher in intestinal metaplasia than superficial (p < 0.001) and atrophic (p < 0.001) gastritis. A 1.5-fold increase in *MYC* expression was detected in 83.3% of intestinal metaplasia samples. The *MYC* mRNA expression tended to be higher in samples with H. pylori than samples without this pathogen (T_53_ = −1.934, p = 0.058, by Student test, r = 0.257). *MYC* and *TP53* mRNA expression were not correlated (p = 0.334, r = 0.133). *hTERT* was weakly correlated to *MYC* and *TP53* mRNA expression (p = 0.047, r = 0.267; p = 0.028, r = 0.296, respectively).

Higher *hTERT* and *MYC* mRNA expression was associated with MYC immunoreactivity (T_53_ = −3.218, p = 0.002, by Student test, r = 0.398; T_53_ = −-7.429, p < 0.001, r = 0.701, respectively). Higher *hTERT, MYC,* and *TP53* mRNA expression was associated with hTERT (T_53_ = −3.658, p = 0.001, r = 0.442; T_53_ = −5.88, p < 0.001, r = 0.621; T_53_ = −2.316, p = 0.024; r = 0.298, respectively) and p53 (T_53_ = −2.495, p = 0.016, r = 0.319; T_53_ = −4.64, p < 0.001, r = 0.530; T_53_ = −2.674, p = 0.010; r = 0.339, respectively) immunoreactivity.

One sample of superficial gastritis presented 3 copies of *TP53* and this case was excluded from the CNV statistical analyses. Loss of *TP53* copies was observed only in the group of intestinal metaplasia samples (Table 1). The frequency of *TP53* loss was significantly higher in intestinal metaplasia samples compared to gastritis specimens (χ^2^ = 6.353, df = 1, p = 0.033, by Fisher exact test, V = 0.343, OR = 2.4). The loss of *TP53* copies was associated with hTERT (χ^2^ = 18.265, df = 1, p = 0.002, V = 0.582; OR = 1.6) and p53 (χ^2^ = 9.926, df = 1, p = 0.03, V = 0.429, OR = 1.47) immunoreactivity. However, this analysis should be considered with care since only 3 cases presented loss of *TP53* copies.

## Discussion

Telomerase activation is thought to be essential for the stabilization of telomere length, through which immortalization and oncogenesis are achieved, but little is known about the regulation of the hTERT subunit in human precancerous gastric lesions. In the present study, we observed that hTERT, MYC, and p53 immunoreactivity was only present in intestinal metaplasia samples. In addition, we detected a strong association among these three proteins and 27.8% of intestinal metaplasia samples presented immunostaining of the three studied proteins. MYC is a transcriptional activator and p53 is a repressor of *hTERT* expression [6]. Some studies favored the view that MYC drove initial proliferation and subsequent differentiation, concomitant with the activation of the p53 G2 checkpoint and also demonstrated that inactivation of the p53-Rb pathway is required for immortalization through overepression of MYC [13,14]. Thus, some cases of intestinal metaplasia may carry short telomeres and due to this telomere dysfunction, MYC stimulates hTERT expression. However, in the absence of genome checkpoint functions (i.e. p53 mutations or *TP53* deletion), this process will favor the proliferation of immortalized cells carrying genetic and epigenetic alterations and tumor initiation.

It is important to note that, although intestinal metaplasia precedes intestinal-type gastric cancer, only few individuals with this preneoplastic lesion will develop gastric tumors. Further investigation are necessary to evaluate the role of hTERT, MYC, and p53 proteins – alterations common described in gastric neoplasia – in the disease progression, ideally with biopsies of intestinal metaplasia and tumor from the same patients. These biomarkers may be useful for the assessment of gastric cancer risk if validated in a larger clinical study sets.

Some studies demonstrated that hTERT expression increases with the sequential steps of intestinal-type gastric carcinogenesis [15-19], suggesting that hTERT deregulation represents an important step in the carcinogenesis progress. We also previously demonstrated that 80% of gastric tumors and no non-neoplastic gastric mucosa of individuals from Northern Brazil presented hTERT immunoreactivity, suggesting that hTERT may have an impact on the anti-telomerase strategy for cancer therapy [20].

The detection of MYC immunoreactivity in intestinal metaplasia of individuals from three Northern Brazil populations corroborates previous studies of our group that demonstrated the presence of MYC protein overexpression only in intestinal metaplasia and neoplastic tissue from all patients with intestinal type gastric cancer, which is preceded by preneoplastic lesions [21-23], as well as in intestinal metaplasia of non-human primates treated with N-methyl-nitrosourea (MNU) [24]. On the other hand, MYC immunoreactivity was described in gastritis samples, as well as intestinal metaplasia, in Asian populations [15,25-27].

Here, MYC immunoreactivity was strongly associated with increased mRNA expression. To our knowledge, this is the first study to sensitively quantify *MYC* mRNA expression in precancerous gastric lesions. The increased MYC expression in intestinal metaplasia supports a previous study of our group in non-human primates, in which we demonstrated a continuous increase of *MYC* mRNA expression during the sequential steps of gastric carcinogenesis in MNU-treated animals [24]. In these animals, the mRNA expression increased about 3-fold in intestinal metaplasia compared to normal gastric mucosa. In addition, we previously reported that a significant increase of *MYC* copy number was seen with the evolution of carcinogenesis process in humans and non-human primates [21,24], which may contribute to the increased mRNA expression and protein immunoreactivity. In addition, *MYC* amplification or trisomy of chromosome 8, where *MYC* is located, was detected in all human gastric cancer of individuals from Northern Brazil [21-23,28-31], as well as in gastric cancer cell lines established from the tumors of Brazilian patients [32-35], supporting that *MYC* has a key role in gastric carcinogenesis.

Previously we also reported the presence of p53 immunoreactivity in all intestinal-type gastric cancer of individuals from Northern Brazil [12]. The p53 immunoreactivity usually depends on the accumulation of mutated p53 proteins in the cell, which leads to a longer half-life [36]. Some studies have demonstrated *TP53* mutations in gastritis [37] and intestinal metaplasia [38,39] as well as gastric cancer, which corroborates the observation of p53 immunoreactivity in intestinal metaplasia samples of the studied population. The presence of p53 immunostaining only in intestinal metaplasia corroborates previous studies of literature [15,40]. However, Targa et al. [41] reported p53 overexpression in 5/19 (26.3%) of chronic gastritis, 1/8 (12.5%) of atrophic gastritis and 2/11 (18.2%) of intestinal metaplasia of individuals from Southeastern Brazil.

To our knowledge, few studies evaluated the number of copies of *TP53* in pre-neoplastic gastric lesions. Loss of heterozygosity at the *TP53* locus is one of the most common mechanisms involved in this gene pathway deregulation. Loss of *TP53* is a frequent finding in gastric cancer [42]. Previously, our group demonstrated that the loss of *TP53* copies and aneusomy of chromosome 17, where this gene is located, was present in all gastric cancer samples of individuals from Pará State, Northern Brazil [12], and also in all gastric cancer cell lines established from neoplasias in this population [33,43]. Here, we observed *TP53* deletion in 16.7% of intestinal metaplasia in individuals from Northern Brazil by qPCR. In a Southeastern Brazilian population, it was described that 3/5 (60%) of intestinal metaplasia samples presented loss of *TP53* by fluorescence in situ hybridization (FISH) assay [36]. Williams et al. [42] also reported that the deletion of *TP53* was a common event in premalignant stages of gastric carcinogenesis by FISH analyses. These authors demonstrated that this alteration was about 3-fold increased in intestinal metaplasia (N = 4) compared to normal gastric tissue. Since *TP53* is a critical tumor suppressor gene in the carcinogenesis process, the loss of this gene copy and its protein immunoreactivity in the intestinal metaplasia stage may contribute for tumor initiation.

## Conclusions

In conclusion, hTERT, MYC, and TP53 are deregulated in intestinal metaplasia of individuals from Northern Brazil and these alterations may facilitate tumor initiation.

## Methods

### Clinical samples

Samples were obtained from cancer-free patients including 19 with superficial gastritis, 18 with atrophic gastritis, and 18 patients with intestinal metaplasia by endoscopy. From each patient, normal tissue sample were also collected. Samples were collected at endoscopy services in Pará, Maranhão and Ceará States in Northern Brazil. Informed consent with approval of the ethics committee of the Federal University of Pará was obtained. Tissue specimens were immediately frozen in liquid nitrogen and kept at −80°C until RNA and DNA extraction. All studied samples were negative for Epstein-Barr infection by in situ hybridization method [44]. The presence of *Helicobacter pylori*, a class I carcinogen, in gastric samples was detected by the PCR assay for the urease gene [45]. Table 1 shows the clinicopathological characteristics of patients.

### Immunohistochemistry

Immunohistochemical analyses were performed on formalin-fixed, paraffin-embedded sections. Immunohistochemical staining was performed on the paraffin sections according to Calcagno et al. [22] with primary mouse monoclonal antibody against hTERT (dilution 1:50; clone 44 F12, Novocastra Laboratories Ltd, UK), MYC (dilution 1:150; sc-40, Santa Cruz Biotechnology, USA and Zymed®, USA), or p53 (dilution 1:50; Dakocytomation, USA). A universal peroxidase-conjugated secondary antibody kit (LSAB System, DakoCytomation, USA) was used for the detection system, and diaminobenzidine (DAB) was the applied chromogen. Positive protein expression was defined as clear nuclear imunostaining in more than 10% of the cells.

### mRNA expression

Total RNA was extracted with Tri-reagent (Applied Biosystems, USA) following the manufacturer’s instructions. RNA concentration and quality were determined using the NanoDrop spectrophotometer (Kisker, Germany) and 1% agarose gels. Complementary DNA was synthesized using High-Capacity cDNA Archive (Applied Biosystems, Poland).

*hTERT*, *MYC,* and *TP53* mRNA expression was evaluated by quantitative reverse transcription PCR (qRT-PCR) with primers and TaqMan probes purchased as Assays-on-demand Products for Gene Expression (Applied Biosystems, USA). *GAPDH* gene was selected as an internal control for RNA input and reverse transcription efficiency. All real-time qRT-PCR reactions were performed in triplicate for all target genes (*hTERT*: Hs00972656_m1; *MYC*: Hs00153408_m1; *TP53*: Hs01034249_m1) and the internal control (*GAPDH*: NM_002046.3).

Relative quantification (RQ) of the gene expression was calculated according to Livak and Schmittgen [46]. In the present study, the corresponding normal tissue sample was designated as a calibrator for superficial gastritis, atrophic gastritis, or intestinal metaplasia sample from each patient.

### *TP53* copy number variation (CNV)

Quantitative TaqMan CNV assays (Applied Biosystems, USA) were used as a confirmation to FISH analysis. Duplex quantitative PCR (qPCR) was performed using the FAM/MGB-labeled TaqMan probe for *TP53* gene (Hs06423639_cn) and VIC/TAMRA-labeled TaqMan CNV *RNAse P* (#4403326) for the internal control. qPCR reactions were performed in quadruplicate with genomic DNA (gDNA) according to the manufacturer's protocol and cycling conditions in 7500 Fast Real-Time PCR (Applied Biosystems, USA). Relative quantification analysis was done to estimate the copy number for each sample by using the Copy Caller Software V1.0 (Applied Biosystems, USA). A known human gDNA (Promega, USA) was used for calibration.

### Data analysis

Chi-square test was performed to analyze hTERT, MYC, and p53 immunostaining, as well as *TP53* CNV data. To take into account the multiple testing, Bonferroni corrections were applied to adjust the Chi-squared p value when necessary. Shapiro-Wilk normality test was used to evaluate the normal distribution of mRNA expression data and to determine subsequent use of appropriate tests for statistical comparison. Data that were not normally distributed were transformed (z-score transformation) for the analysis of variance in gene expression such that they followed a normal distribution. Analyses of variance in mRNA expression were performed by one-way ANOVA followed by Tukey (homogeneity of variances according to Levene test) post-hoc test. Chi-square and Student’s T test were used to assess the relationship between gene expression, protein immunoreactivity, and CNV results and clinicopathological factors.

The effect size for Chi-square was based on Cramer’s phi (V) and the effect size correlation directly from the Student T test as “r” (Pearson correlation coefficient), in which a value between 0.1-0.29 was determined as a small effect size; 0.3-0.49 as a medium effect size; and 0.50 or above as a large effect size. The effect size for ANOVA analyses was based on Eta Squared (η^2^), in which values 0.15 and below were determined as a small effect size; 0.16-0.40 as a medium effect size; and above 0.40 as a large effect size.

The correlation between *hTERT*, *MYC,* and *TP53* mRNA expression was analyzed by the Pearson test, in which a value below 0.30 was determined as a weak correlation; 0.30-0.70 as a medium correlation; and above 0.70 as a strong correlation.

In all analyses, the confidence interval was 95% and *p* values less than 0.05 were considered significant.

## Abbreviations

CNV: Copy Number Variation; DAB: diaminobenzidine; gDNA: genomic DNA; MNU: Nmethyl-nitrosourea; qPCR: quantitative PCR; qRT-PCR: quantitative reverse transcription PCR; RQ: Relative Quantification.

## Competing interests

All authors declare that they have no conflicts of interest.

## Authors’ contributions

MFL, TCRS, PPA, MACS and RRB conceived and designed the experiments. TCRS, MFL, DQC, RCM and MQN performed the experiments. TCRS, MFL, DQC, CRT, ASK, NPCS, MACS, and RRB analyzed the data. NPCS, SHBR and PPA contributed reagents/materials/analysis tools. MFL, TCRS, MACS and RRB wrote the paper. All authors read and approved the final manuscript.

## Pre-publication history

The pre-publication history for this paper can be accessed here:

http://www.biomedcentral.com/1471-230X/12/85/prepub

## References

[B1] Jemal A Bray F Center MM Ferlay J Ward E Forman D Global cancer statistics CA Cancer J Clin 2011 61 2 69 90 10.3322/caac.20107 21296855

[B2] Instituto Nacional de Câncer - INCA Estimate/2010 - Incidence of Cancer in Brazil 2009 Rio de JaneiroL: Instituto Nacional de Câncer/Ministério da Saúde 98

[B3] Correa P Human gastric carcinogenesis: a multistep and multifactorial process–First American Cancer Society Award Lecture on Cancer Epidemiology and Prevention Cancer Res 1992 52 24 6735 6740 1458460

[B4] Kim NW Piatyszek MA Prowse KR Harley CB West MD Ho PL Coviello GM Wright WE Weinrich SL Shay JW Specific association of human telomerase activity with immortal cells and cancer Science 1994 266 5193 2011 2015 10.1126/science.7605428 7605428

[B5] Artandi SE DePinho RA Mice without telomerase: what can they teach us about human cancer? Nat Med 2000 6 8 852 855 10.1038/78595 10932211

[B6] Kyo S Takakura M Fujiwara T Inoue M Understanding and exploiting hTERT promoter regulation for diagnosis and treatment of human cancers Cancer Sci 2008 99 8 1528 1538 10.1111/j.1349-7006.2008.00878.x 18754863 PMC11158053

[B7] Calcagno DQ Leal MF Assumpcao PP Smith MA Burbano RR MYC and gastric adenocarcinoma carcinogenesis World J Gastroenterol 2008 14 39 5962 5968 10.3748/wjg.14.5962 18932273 PMC2760197

[B8] Fernandez PC Frank SR Wang L Schroeder M Liu S Greene J Cocito A Amati B Genomic targets of the human c-Myc protein Genes Dev 2003 17 9 1115 1129 10.1101/gad.1067003 12695333 PMC196049

[B9] Dang CV O'Donnell KA Zeller KI Nguyen T Osthus RC Li F The c-Myc target gene network Semin Cancer Biol 2006 16 4 253 264 10.1016/j.semcancer.2006.07.014 16904903

[B10] Szymanska K Hainaut P TP53 and mutations in human cancer Acta Biochim Pol 2003 50 1 231 238 12673364

[B11] Vousden KH Ryan KM p53 and metabolism Nat Rev Cancer 2009 9 10 691 700 10.1038/nrc2715 19759539

[B12] Khayat AS Guimaraes AC Calcagno DQ Seabra AD Lima EM Leal MF Faria MH Rabenhorst SH Assumpcao PP Demachki S Interrelationship between TP53 gene deletion, protein expression and chromosome 17 aneusomy in gastric adenocarcinoma BMC Gastroenterol 2009 9 55 10.1186/1471-230X-9-55 19619279 PMC2716360

[B13] Pelengaris S Rudolph B Littlewood T Action of Myc in vivo - proliferation and apoptosis Curr Opin Genet Dev 2000 10 1 100 105 10.1016/S0959-437X(99)00046-5 10679391

[B14] Dazard JE Piette J Basset-Seguin N Blanchard JM Gandarillas A Switch from p53 to MDM2 as differentiating human keratinocytes lose their proliferative potential and increase in cellular size Oncogene 2000 19 33 3693 3705 10.1038/sj.onc.1203695 10949923

[B15] Lan J Xiong YY Lin YX Wang BC Gong LL Xu HS Guo GS Helicobacter pylori infection generated gastric cancer through p53-Rb tumor-suppressor system mutation and telomerase reactivation World J Gastroenterol 2003 9 1 54 58 12508351 10.3748/wjg.v9.i1.54 PMC4728249

[B16] Wang W Luo HS Yu BP Expression of NF-kappaB and human telomerase reverse transcriptase in gastric cancer and precancerous lesions World J Gastroenterol 2004 10 2 177 181 14716817 10.3748/wjg.v10.i2.177 PMC4716998

[B17] Cassaro M Rugge M Tieppo C Giacomelli L Velo D Nitti D Farinati F Indefinite for non-invasive neoplasia lesions in gastric intestinal metaplasia: the immunophenotype J Clin Pathol 2007 60 6 615 621 10.1136/jcp.2006.040386 17557866 PMC1955067

[B18] Gulmann C Lantuejoul S Grace A Leader M Patchett S Kay E Telomerase activity in proximal and distal gastric neoplastic and preneoplastic lesions using immunohistochemical detection of hTERT Dig Liver Dis 2005 37 6 439 445 10.1016/j.dld.2005.01.008 15893283

[B19] Jong HS Park YI Kim S Sohn JH Kang SH Song SH Bang YJ Kim NK Up-regulation of human telomerase catalytic subunit during gastric carcinogenesis Cancer 1999 86 4 559 565 10.1002/(SICI)1097-0142(19990815)86:4<559::AID-CNCR3>3.0.CO;2-4 10440682

[B20] Gigek CO Leal MF Silva PN Lisboa LC Lima EM Calcagno DQ Assumpcao PP Burbano RR Smith MA hTERT methylation and expression in gastric cancer Biomarkers 2009 14 8 630 636 10.3109/13547500903225912 20001710

[B21] Calcagno DQ Leal MF Demachki S Araujo MT Freitas FW SD Oliveira e, Assumpcao PP, Ishak G, de Arruda Cardoso Smith M, Burbano RR: MYC in gastric carcinoma and intestinal metaplasia of young adults Cancer Genet Cytogenet 2010 202 1 63 66 10.1016/j.cancergencyto.2010.05.020 20804924

[B22] Calcagno DQ Leal MF Seabra AD Khayat AS Chen ES Demachki S Assumpcao PP Faria MH Rabenhorst SH Ferreira MV Interrelationship between chromosome 8 aneuploidy, C-MYC amplification and increased expression in individuals from northern Brazil with gastric adenocarcinoma World J Gastroenterol 2006 12 38 6207 6211 17036397 10.3748/wjg.v12.i38.6207 PMC4088119

[B23] Costa Raiol LC Figueira Silva EC Mendes da Fonseca D Leal MF Guimaraes AC Calcagno DQ Khayat AS Assumpcao PP de Arruda Cardoso Smith M Burbano RR Interrelationship between MYC gene numerical aberrations and protein expression in individuals from northern Brazil with early gastric adenocarcinoma Cancer Genet Cytogenet 2008 181 1 31 35 10.1016/j.cancergencyto.2007.10.011 18262050

[B24] da Costa Jde F Leal MF Silva TC Andrade Junior EF Rezende AP Muniz JA Lacreta Junior AC Assumpcao PP Calcagno DQ Demachki S Experimental gastric carcinogenesis in Cebus apella nonhuman primates PLoS One 2011 6 7 e21988 10.1371/journal.pone.0021988 21811552 PMC3140998

[B25] Han JC Zhang KL Chen XY Jiang HF Kong QY Sun Y Wu ML Huang L Li H Liu J Expression of seven gastric cancer-associated genes and its relevance for Wnt, NF-kappaB and Stat3 signaling APMIS 2007 115 12 1331 1343 10.1111/j.1600-0643.2007.00695.x 18184402

[B26] Yang GF Deng CS Xiong YY Gong LL Wang BC Luo J Expression of nuclear factor-kappa B and target genes in gastric precancerous lesions and adenocarcinoma: association with Helicobactor pylori cagA (+) infection World J Gastroenterol 2004 10 4 491 496 14966904 10.3748/wjg.v10.i4.491 PMC4716967

[B27] Zhang GX Gu YH Zhao ZQ Xu SF Zhang HJ Wang HD Hao B Coordinate increase of telomerase activity and c-Myc expression in Helicobacter pylori-associated gastric diseases World J Gastroenterol 2004 10 12 1759 1762 15188501 10.3748/wjg.v10.i12.1759 PMC4572264

[B28] Assumpcao PP Ishak G Chen ES Takeno SS Leal MF Guimaraes AC Calcagno DQ Khayat AS Demachki S Smith MA Numerical aberrations of chromosome 8 detected by conventional cytogenetics and fluorescence in situ hybridization in individuals from northern Brazil with gastric adenocarcinoma Cancer Genet Cytogenet 2006 169 1 45 49 10.1016/j.cancergencyto.2006.03.019 16875936

[B29] Burbano RR Assumpcao PP Leal MF Calcagno DQ Guimaraes AC Khayat AS Takeno SS Chen ES De Arruda Cardoso Smith M C-MYC locus amplification as metastasis predictor in intestinal-type gastric adenocarcinomas: CGH study in Brazil Anticancer Res 2006 26 4B 2909 2914 16886612

[B30] Calcagno DQ Leal MF Taken SS Assumpcao PP Demachki S Smith Mde A Burbano RR Aneuploidy of chromosome 8 and C-MYC amplification in individuals from northern Brazil with gastric adenocarcinoma Anticancer Res 2005 25 6B 4069 4074 16309200

[B31] Calcagno DQ Guimaraes AC Leal MF Seabra AD Khayat AS Pontes TB Assumpcao PP De Arruda Cardoso Smith M, Burbano RR: MYC insertions in diffuse-type gastric adenocarcinoma Anticancer Res 2009 29 7 2479 2483 19596917

[B32] Leal MF Calcagno DQ da Borges Costa JF, Silva TC, Khayat AS, Chen ES, Assumpcao PP, de Arruda Cardoso Smith M, Burbano RR: MYC, TP53, and chromosome 17 copy-number alterations in multiple gastric cancer cell lines and in their parental primary tumors J Biomed Biotechnol 2011 2011 631268 21528007 10.1155/2011/631268 PMC3082130

[B33] Leal MF Nascimento JL Martins do, da Silva CE, Vita Lamarao MF, Calcagno DQ, Khayat AS, Assumpcao PP, Cabral IR, de Arruda Cardoso Smith M, Burbano RR: Establishment and conventional cytogenetic characterization of three gastric cancer cell lines Cancer Genet Cytogenet 2009 195 1 85 91 10.1016/j.cancergencyto.2009.04.020 19837275

[B34] Ribeiro HF Alcantara DF Matos LA Sousa JM Leal MF Smith MA Burbano RR Bahia MO Cytogenetic characterization and evaluation of c-MYC gene amplification in PG100, a new Brazilian gastric cancer cell line Braz J Med Biol Res 2010 43 8 717 721 10.1590/S0100-879X2010007500068 20658094

[B35] Costa Guimaraes A Goncalves Quintana L Ferreira Leal M Satomi Takeno S Pimentel Assumpcao P Moura Lima E Salim Khayat A Suchi Chen E de Arruda Cardoso Smith M Rodriguez Burbano R Aneuploidy of chromosome 8 detected by fluorescence in situ hybridisation in ACP01 cell line gastric adenocarcinoma Clin Exp Med 2006 6 3 129 133 10.1007/s10238-006-0108-5 17061062

[B36] Cesar AC Borim AA Caetano A Cury PM Silva AE Aneuploidies, deletion, and overexpression of TP53 gene in intestinal metaplasia of patients without gastric cancer Cancer Genet Cytogenet 2004 153 2 127 132 10.1016/j.cancergencyto.2004.01.017 15350302

[B37] Morgan C Jenkins GJ Ashton T Griffiths AP Baxter JN Parry EM Parry JM Detection of p53 mutations in precancerous gastric tissue Br J Cancer 2003 89 7 1314 1319 10.1038/sj.bjc.6601302 14520466 PMC2394306

[B38] Uchino S Noguchi M Ochiai A Saito T Kobayashi M Hirohashi S p53 mutation in gastric cancer: a genetic model for carcinogenesis is common to gastric and colorectal cancer Int J Cancer 1993 54 5 759 764 10.1002/ijc.2910540509 8392033

[B39] Shiao YH Rugge M Correa P Lehmann HP Scheer WD p53 alteration in gastric precancerous lesions Am J Pathol 1994 144 3 511 517 8129036 PMC1887105

[B40] Wang J Chi DS Kalin GB Sosinski C Miller LE Burja I Thomas E Helicobacter pylori infection and oncogene expressions in gastric carcinoma and its precursor lesions Dig Dis Sci 2002 47 1 107 113 10.1023/A:1013223722331 11837709

[B41] Targa AC Cesar AC Cury PM Silva AE Apoptosis in different gastric lesions and gastric cancer: relationship with Helicobacter pylori, overexpression of p53 and aneuploidy Genet Mol Res 2007 6 3 554 565 17985308

[B42] Williams L Jenkins GJ Doak SH Fowler P Parry EM Brown TH Griffiths AP Williams JG Parry JM Fluorescence in situ hybridisation analysis of chromosomal aberrations in gastric tissue: the potential involvement of Helicobacter pylori Br J Cancer 2005 92 9 1759 1766 10.1038/sj.bjc.6602533 15827559 PMC2362026

[B43] Leal MF Calcagno DQ da Costa JF Borges, Silva TC, Khayat AS, Chen ES, Assumpcao PP, de Arruda Cardoso Smith M, Burbano RR: MYC, TP53, and chromosome 17 copy-number alterations in multiple gastric cancer cell lines and in their parental primary tumors J Biomed Biotechnol 2010 2011 631268 21528007 10.1155/2011/631268 PMC3082130

[B44] Bacchi MM Bacchi CE Alvarenga M Miranda R Chen YY Weiss LM Burkitt's lymphoma in Brazil: strong association with Epstein-Barr virus Mod Pathol 1996 9 1 63 67 8821959

[B45] Clayton CL Kleanthous H Coates PJ Morgan DD Tabaqchali S Sensitive detection of Helicobacter pylori by using polymerase chain reaction J Clin Microbiol 1992 30 1 192 200 1734052 10.1128/jcm.30.1.192-200.1992 PMC265019

[B46] Arocho A Chen B Ladanyi M Pan Q Validation of the 2-DeltaDeltaCt calculation as an alternate method of data analysis for quantitative PCR of BCR-ABL P210 transcripts Diagn Mol Pathol 2006 15 1 56 61 10.1097/00019606-200603000-00009 16531770

